# Pertussis Surveillance in Private Pediatric Practices, France, 2002–2006

**DOI:** 10.3201/eid1407.071246

**Published:** 2008-07

**Authors:** Nicole Guiso, France de La Rocque, Elisabeth Njamkepo, Aurelie Lécuyer, Corinne Levy, Olivier Romain, Franck Thollot, Véronique Abitbol, Benoit Soubeyrand, Robert Cohen, the French Pediatrics Groups Association Clinique et Thérapeutique Infantile du Val de Marne, Association Française de Pédiatrie Ambulatoire

**Affiliations:** * Institut Pasteur, Paris, France; † Association Clinique et Thérapeutique Infantile du Val de Marne, Saint Maur des Fossés, France; ‡ Association Française de Pédiatrie Ambulatoire, Besançon, France; § GlaxoSmithKline, Marly le Roi, France; ¶ Sanofi Pasteur MSD, Lyon, France; # Centre Hospitalier Intercommunal de Créteil, Créteil, France

**Keywords:** Pertussis epidemiology, pertussis surveillance, whole-cell pertussis vaccine, France, letter

**To the Editor:** In France, pertussis epidemiology has been extensively studied since 1993. Immunization of children with a highly efficacious pertussis whole-cell (Pw) vaccine (Sanofi Pasteur MSD, Lyon, France) for 40 years (since 1966) has reduced the incidence of pertussis. It has been demonstrated that infectious or vaccinal immunity to pertussis wanes with time and that pertussis is no longer a pediatric disease (*1*–*5*). Transmission now occurs predominantly from adolescents and adults to unvaccinated newborns.

From 1966 through 1995, primary vaccination against pertussis was administered to children at 3, 4, and 5 months of age, and a booster was given at ≈2 years of age. Since 1995, primary vaccination has been administered at 2, 3, and 4 months of age, and a booster is given at 16–18 months of age. Duration of protection of children immunized with Pw vaccine at these schedules is estimated to be ≈7–9 years (*1*,*5*).

In response to the problem of waning immunity, a second pertussis booster immunization at 11–13 years of age was introduced in 1998 (*6*). Development of pertussis acellular (Pa) vaccines has enabled administration of this booster immunization. The French hospital network surveillance system (Renacoq) was established in 1996 to monitor severe pertussis in infants and the effect of late booster immunizations. A cyclic disease pattern was observed; peaks were noted for 1993, 1997, 2000, and 2005. However, the last peak had a low amplitude; since then a diminution in the proportion of siblings who transmitted the infection to young infants was observed (*2*). These results could have been caused by adolescent booster immunizations.

We evaluated whether the duration of immunity induced by Pw vaccine was still similar to the duration estimated in 1993–1994. This surveillance was necessary because antigenic changes in circulating isolates of *Bordetella pertussis* were observed when compared with vaccine strains (*7*). To achieve this goal, a private pediatric network was set up and data from this surveillance are presented.

From September 2002 through April 2006, 79 pediatricians in France enrolled all infants and children suspected of having pertussis. A standardized data form was completed for age, sex, vaccination data, and source of infection. Biologic confirmation of cases was obtained by using routine laboratory diagnoses, i.e., culture, PCR, or serology. Real-time PCR was performed according to consensus rules (*8*). Routine serodiagnosis was performed by using purified pertussis toxin and Western blotting according to the method of Guiso et al. (*9*) because this is the only diagnostic test free for patients in France. Serologic diagnosis was made by detecting antibodies to pertussis toxin in unvaccinated children or in those vaccinated >1 year earlier. Epidemiologic case-patients were defined as those with a cough for 14 days who had contacts with a confirmed case-patient within 4 weeks of the onset of the cough. No confirmed suspected case-patients had coughs; all were negative for pertussis by biologic diagnosis and did not report contact with a confirmed case-patient.

A total of 383 children were enrolled in the study. However, vaccination status and a biologic diagnosis were available for only 139 children (Table). Forty-seven children had biologically confirmed cases and 92 had nonconfirmed cases. Among children with confirmed cases, only 22 had been vaccinated. At time of disease, the mean ± SD age of these children was 9.9 ± 2.1 years. This age was similar to the age observed during 1993–1994 (*1*,*5*).

**Table:** Characteristics of confirmed and nonconfirmed pertussis case-patients for whom vaccination status with pertussis whole-cell (Pw) vaccine was known

Characteristic	Vaccinated 4× with Pw vaccine (N = 70)			Unvaccinated (N = 69)		
	Confirmed case-patients (n = 22)	Nonconfirmed case-patients (n = 48)		Confirmed case-patients (n = 25)		Nonconfirmed case-patients (n = 44)
Diagnostic method						
Culture	0†			3*		
PCR	7†			17*		
Serology	13			3		
Epidemiology	2			2		
Age, y	9.9 ± 2.1†	7.8 ± 3.4		2.3 ± 3.9†		0.8 ± 2.3
p value for age	0.008			0.0046		
Source of contamination						
Adults	5			7		
Adolescents	4			5		
Infants	1 (unvaccinated)			0		

*p = 0.0009 for PCR and culture for case-patients receiving 4 doses of Pw vaccine versus unvaccinated confirmed case-patients. †p<0.0001 by age for case-patients receiving 4 doses of Pw vaccine versus unvaccinated confirmed case-patients.

The diagnosis for the 92 children suspected of having pertussis was not confirmed biologically. Culture and PCR are used for diagnosis early in the course of pertussis. However, serologic analysis is used later because antibodies are rarely detected before 3 weeks of onset of a cough. More culture and PCR diagnoses were performed for unvaccinated confirmed case-patients than for vaccinated confirmed case-patients. This finding suggests that unvaccinated children were seen by their pediatricians earlier than vaccinated children because the disease was less severe in vaccinated children or that vaccinated children were older than unvaccinated children.

The source of contamination was known for 47% of the confirmed case-patients (Table). This source was either adults (54.4%) or adolescents (41%) who did not receive their second booster immunization or an unvaccinated infant (4.5%). These data are similar to those obtained by the French hospital-based surveillance (*2*). They also support the strategy started in 2004 of recommending a pertussis booster immunization for adults in contact with children and all healthcare workers who come in contact with infants (*10*)

In conclusion, this pediatric surveillance confirms the usefulness of following vaccine recommendations for pertussis and of using biologic techniques to confirm a diagnosis. The vaccine strategy recommending a booster vaccination at 11–13 years of age is still in accordance with epidemiologic features observed. Pediatricians should continue this surveillance to evaluate evolution of *B. pertussis* populations and the effect of replacing Pw vaccines with Pa vaccines.
